# A case with an ectopic ejaculatory duct opening into the bladder trigone in Zinner syndrome, congenital unilateral renal agenesis, and an ipsilateral seminal vesicle cyst

**DOI:** 10.1002/iju5.12763

**Published:** 2024-07-30

**Authors:** Taiju Hyuga, Kazuya Tanabe, Taro Kubo, Kimihiko Moriya

**Affiliations:** ^1^ Department of Pediatric Urology Jichi Medical University, Children's Medical Center Tochigi Shimotsuke Tochigi Japan

**Keywords:** ectopic ejaculatory duct opening, seminal vesicle cyst, Zinner syndrome

## Abstract

**Introduction:**

This report describes a case with an ectopic ejaculatory duct opening into the bladder trigone in Zinner syndrome, congenital unilateral renal agenesis, and an ipsilateral seminal vesicle cyst.

**Case presentation:**

The patient was identified when no left kidney was detected in the fetal period. Abdominal ultrasonography and pelvic plain MRI at 6 months old revealed a 10‐mm cystic lesion on the dorsal aspect of the bladder. Cysto‐urethroscopy at 1 year old revealed a rather short posterior urethra and right and left inferior crests extending from the posterior urethra beyond the bladder neck. The ejaculatory duct opening was identified on the bladder trigone.

**Conclusion:**

Anatomical abnormality of the ejaculatory duct may represent a cause of infertility and ejaculatory dysfunction in Zinner syndrome. Endoscopic evaluation should be performed for this rare anomaly, even in children.

**Abbreviations & Acronyms**
DMSA: dimercaptosuccinic acid
LUTS: lower urinary tract symptoms
MRI: magnetic resonance imaging
VCUG: voiding cystourethrography

**Keynote message**

This report describes a case with an ectopic ejaculatory duct opening into the bladder trigone in Zinner syndrome, congenital unilateral renal agenesis, and an ipsilateral seminal vesicle cyst. Anatomical abnormality of the ejaculatory duct may represent a cause of infertility and ejaculatory dysfunction. Endoscopic evaluation should be performed for this rare anomaly, even in children.

## Introduction

The ejaculatory duct originates from the mesonephric duct, shifts distally, then opens into the posterior urethra (prostatic urethra). At ejaculation, semen enters the posterior urethra and is ejaculated through the contractile activity of the internal urethral sphincter. Abnormality of the ejaculatory duct may thus represent a cause of infertility or ejaculatory dysfunction.

Although several abnormalities of the opening of the ejaculatory duct have been reported, an opening proximal to the internal urethral sphincter is very rare.^1^, ^2^, ^3^ We describe a case in which an ectopic ejaculatory duct opened into the bladder trigone in a case of Zinner syndrome, along with congenital unilateral renal agenesis and an ipsilateral seminal vesicle cyst.

## Case presentation

**Fig. 1:**
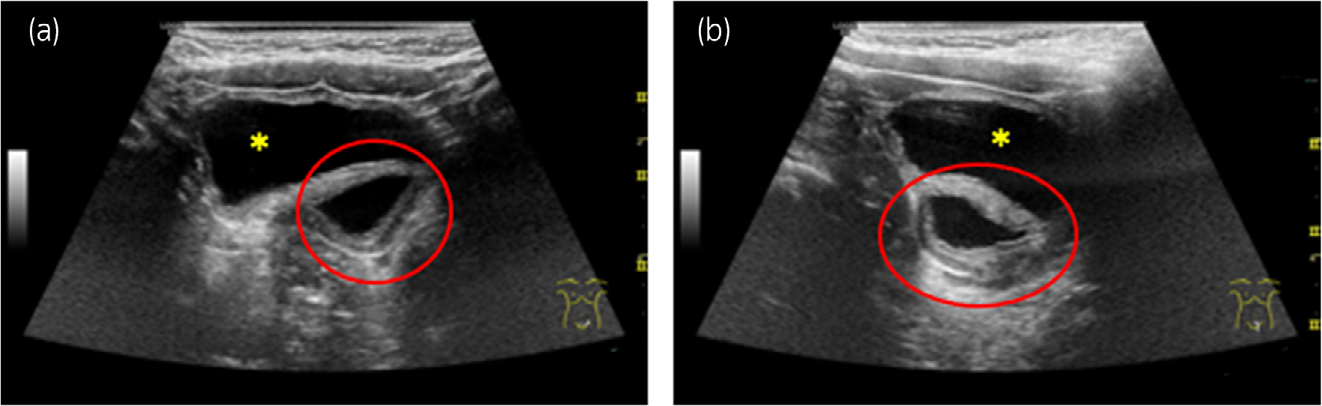
Abdominal ultrasonography. (a) Axial section; (b) sagittal section. Abdominal ultrasonography shows a 10‐mm hypoechoic lesion (red circles) on the left dorsal aspect of the bladder. The bladder is indicated by an asterisk.

**Fig. 2:**
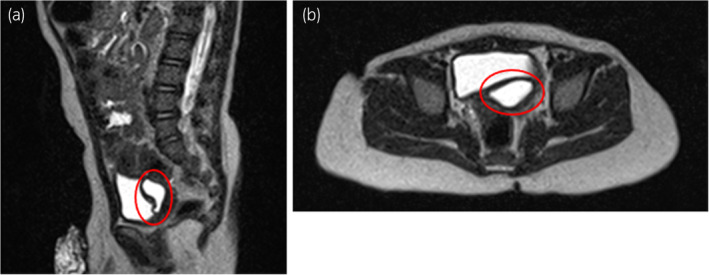
T2‐weighted MRI. (a) A 10‐mm low‐intensity lesion (red circles) is seen on the dorsal side of the bladder in sagittal section. (b) A low‐intensity lesion is apparent on the left side in axial section.

**Fig. 3:**
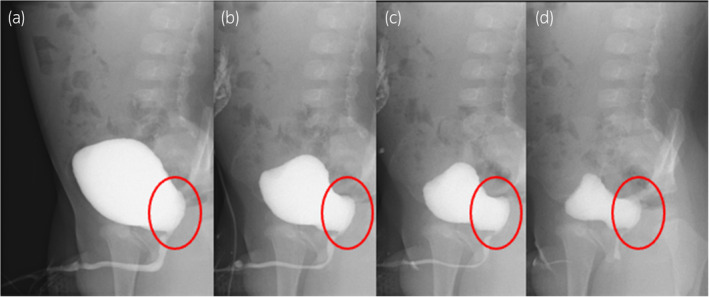
Voiding cystourethrography. In the voiding phase, as voiding process (a–d), the dorsal side of the bladder shows double contrast, attributed to inflow of contrast agent into a diverticular‐like area.

The patient was born at 41 weeks of gestation, weighing 3600 g at birth. The left kidney had been undetectable on ultrasonography or MRI during the fetal period. At 6 months of age, abdominal ultrasonography revealed a 10‐mm cystic lesion on the dorsal position of the bladder, and the patient was referred to our department at 9 months old (Fig. 1). Pelvic plain MRI showed a 10‐mm high intensity lesion on the left and dorsal side of the bladder on T2‐weighted images (Fig. 2). In the voiding phase of VCUG, the dorsal side of the bladder showed double contrast, which was suspected to represent inflow of contrast agent into a diverticular‐like area (Fig. 3). A DMSA renal scan did not visualize a left kidney. Based on these findings, the patient was considered to have a right solitary kidney, and the cystic lesion on the dorsal side of the bladder was considered to represent the remnant Wolffian duct.

**Fig. 4:**
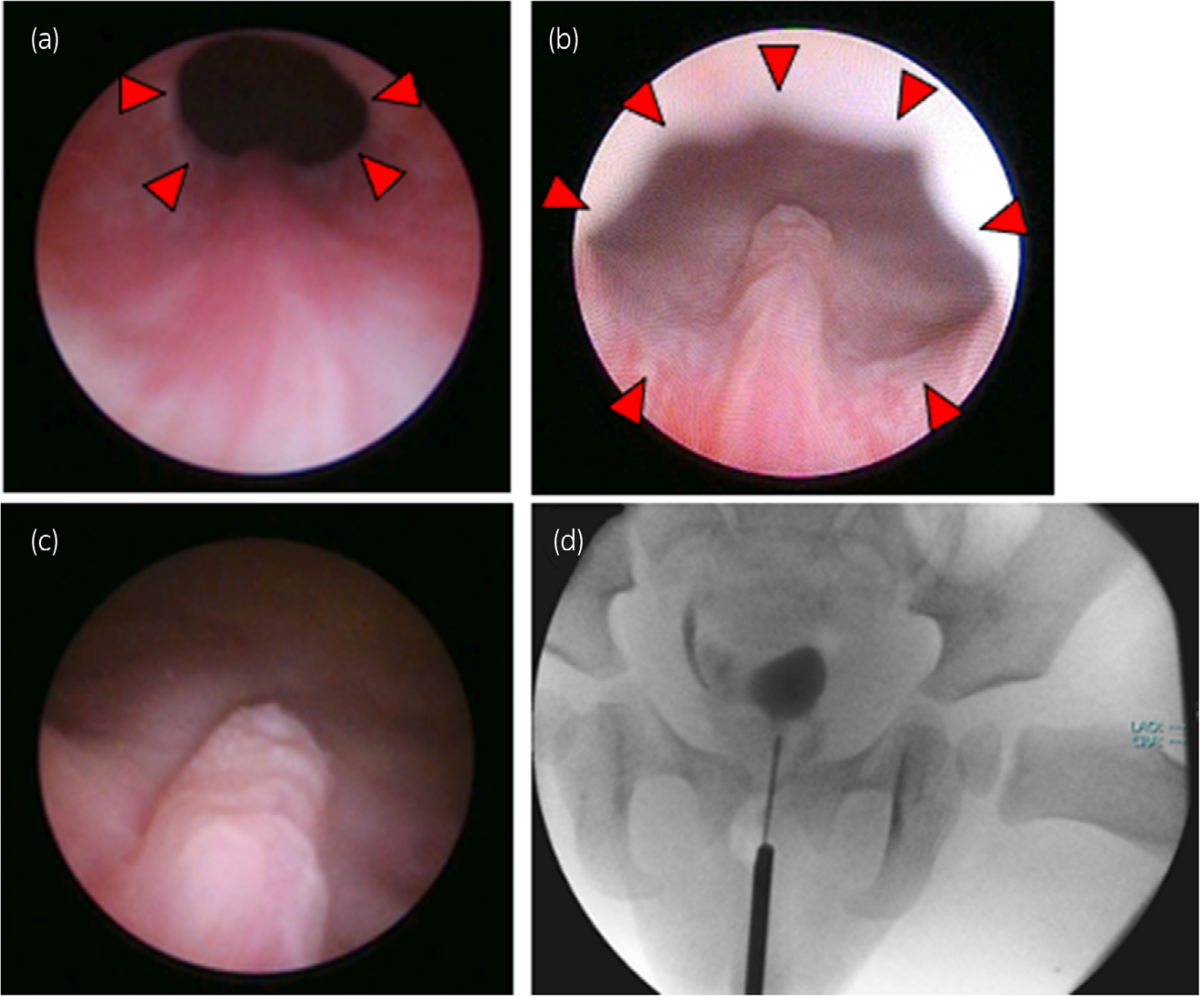
Cystourethroscopic findings and retrograde imaging. (a) Observation of the posterior urethra reveals the right and left inferior crests extending from caudally to cranially. (b, c) The inferior crest extends into the bladder trigone, where the ejaculatory duct opening is identified in (b) distal side and (c) proximal side. (d) A 3‐Fr. ureteral catheter inserted into the left ejaculatory duct opening allows contrast medium to fill the cystic lesion. The bladder neck is indicated by red arrowheads.

Cystoscopy using a 9.5‐Fr cysto‐urethroscope revealed a rather short posterior urethra (Fig. 4a) and right and left inferior crests extending from the posterior urethra beyond the bladder neck, merging to form the ejaculatory duct opening into the bladder trigone (Fig. 4b,c). The bladder trigone was presented the structure of right hemi‐trigone, and the left ureteral orifice was not revealed. The right ureteral orifice was located at the normal position. The ejaculatory duct was opened at the center of the trigon close to the ureteral ridge. Retrograde imaging using a 3‐Fr ureteral catheter inserted through the ejaculatory duct opening showed a cystic lesion on the posterolateral aspect of the bladder (Fig. 4d). In endoscopic examination, the structure of verumontanum was located beyond bladder neck. The contrast imaging through the opening site located at the verumontanum was revealed with laterality. MRI showed the similar findings, so we diagnosed by these findings that the seminal tract was opened in this site. We diagnosed Zinner syndrome, which involves a combination of a seminal vesicle cyst and ipsilateral congenital renal agenesis, with an ectopic ejaculatory duct opening on the bladder trigone.

## Discussion

In this case, we identified an ectopic ejaculatory duct opening into the bladder trigone in Zinner syndrome. Congenital unilateral renal agenesis with ipsilateral seminal vesicle cyst has been reported as Zinner syndrome, which shows an incidence of only 0.00214–0.0046%.^4^, ^5^ Reasons for detection vary, with some cases diagnosed in adulthood due to refractory LUTS or infertility, while others are diagnosed after incidental findings in childhood.^6^ One report estimated that 20% of patients diagnosed with congenital solitary kidney by 20 years old will be diagnosed with Zinner syndrome in the future.^7^ Sixty percent of patients diagnosed with Zinner syndrome experience clinical symptoms, including LUTS, and two‐thirds of symptomatic patients show abnormal uroflowmetry curve patterns.^7^ Larger seminal cyst size correlates significantly with greater LUTS.^7^ Screening for Zinner syndrome is thus recommended for cases with solitary kidney or refractory LUTS in males.

Cases showing ectopic ejaculatory duct opening, particularly with the opening located in the bladder, are quite rare. Most of the limited number of case reports describing ectopic ejaculatory duct opening have been detected in adulthood, usually due to infertility or hematospermia.^3^ Only two pediatric cases with isolated ectopic ejaculatory duct opening have been reported in the literature. The chief complaint in both cases was recurrent epididymitis.^2^, ^8^ The case of Zinner syndrome combined the ectopic ejaculatory duct opening was rare. Only one case of ejaculatory duct ectopic opening combined with Zinner syndrome has been reported previously.^9^ Accordingly, this represents the second report of Zinner syndrome with ectopic opening of the ejaculatory duct. Because of the risks of infertility or ejaculatory dysfunction, routine cystoscopic evaluation is recommended when Zinner syndrome is diagnosed, even in children. We considered to be two benefits of early examination for the patient. First, early treatment might be possible to be performed if onset of symptoms. Second, with the knowledge of his medical status, the early presentation of the infertility risk and early reproductive management is possible in the future.

Embryologically, the ureter sprouts as a ureteric bud from the mesonephric duct, with the ureteric inlet rising cephalad. The ejaculatory duct moves caudally to join the urethra and form the semen tract.^10^ Zinner syndrome results from a developmental anomaly of the unilateral mesonephric duct, leading to a unilateral seminal vesicle cyst and renal agenesis. In the current case, no structure of an ejaculatory duct was identified in the posterior urethra. This finding suggests that caudal migration of the contralateral mesonephric duct may be disturbed by a contralateral developmental anomaly, although the mechanisms of crosstalk between mesonephric ducts on each side during caudal migration remain unknown.

## Conclusion

We have reported a case with ectopic ejaculatory duct opening into the bladder trigone in Zinner syndrome. Since anatomical abnormality of the ejaculatory duct may be one cause of infertility and ejaculatory dysfunction, endoscopic evaluations are warranted when Zinner syndrome is diagnosed. Long‐term follow‐up, including post‐pubertal care, is also necessary.

## Author contributions

Taiju Hyuga: Conceptualization; investigation; methodology; visualization; writing – original draft; writing – review and editing. Kazuya Tanabe: Writing – review and editing. Taro Kubo: Writing – review and editing. Kimihiko Moriya: Supervision; writing – review and editing.

## Conflict of interest

The authors declare no conflict of interest.

## Approval of the research protocol by an Institutional Reviewer Board

Not applicable.

## Informed consent

Informed consent was obtained from the patient's family.

## Registry and the Registration No. of the study/trial

Not applicable.
